# STING-associated vasculopathy with onset in infancy: new clinical findings and mutation in three Turkish children

**DOI:** 10.1186/1546-0096-13-S1-O85

**Published:** 2015-09-28

**Authors:** F Kara Eroglu, I Gursel, M Gursel, A Duzova, AA de Jesus, RT Goldbach-Mansky, S Ozen

**Affiliations:** 1 Hacettepe University, Faculty of Medicine, Pediatric Nephrology-Rheumatology, Ankara, Turkey; 2 Bilkent University, Molecular Biology and Genetics, Ankara, Turkey; 3 Middle East Technical University, Molecular Biology and Genetics, Ankara, Turkey; 4 NIH, Translational Autoinflammatory Disease Section, Bethesda, MD, USA

## Objective

STING-associated vasculopathy with onset in infancy (SAVI) is a recently identified autoinflammatory disease caused by gain-of-function mutations in *TMEM173*. This syndrome is a new interferonopathy characterized by neonatal-onset systemic inflammation with a severe cutaneous vasculopathy leading to extensive tissue loss and interstitial lung disease.

## Patients

We clinically evaluated three patients with acral necrosis and systemic inflammation from three unrelated nonconsangineous Turkish families. Genetic analysis of TMEM173 was performed by direct sequencing.

## Results

Case 1 was a 17 year old boy who presented with tachypnea in infancy and had cold-induced acral necrosis of fingers, toes and ear. His skin biopsy revealed *Periodic acid*-*Schiff* (*PAS*) positive fibrin thrombi in the lumen of ectatic vessels beneath the epidermis. The vessel walls also had PAS-positive thickening, immunofluorescence staining showed fibrinogen and C3. He was initially diagnosed with probable cryofibrinogenemia due to the presence of serum cryofibrinogen in one test. On follow up he had features of interstitial lung disease. His mutation analysis revealed an N154S mutation. Case 2 was a 14 year old girl presenting with joint contracture since infancy, cold induced gangrene of fingers, toes and recurrent sinusitis and cellulitis. Because of high transaminase levels, a liver biopsy was done revealing inflammation, hepatosteatosis and focal fibrosis. Paranasal sinus tomography showed extensive opacification of sinuses and left maxillary sinus medial wall defect. Case 3 was a 17 year old boy presented with cold induced ischemic acral lesions since 3 years of age and progressive spasticity of lower limbs since 12 years of age. Brain CT had showed basal ganglia calcification. Mutation analysis of case 2 and 3 revealed a novel compound heterozygous mutation of V155E/L170Q in TMEM173.

## Conclusion

These three cases widen genetic and clinical features of SAVI syndrome. Besides the vascular ischemic lesions, cranial involvement resembling Aicardi-Goutiers syndrome is a new feature of SAVI syndrome.

## Consent to publish

Written informed consent for publication of their clinical details was obtained from the patient/parent/guardian/relative of the patient.

